# Development and web deployment of an automated neuroradiology MRI protocoling tool with natural language processing

**DOI:** 10.1186/s12911-021-01574-y

**Published:** 2021-07-12

**Authors:** Yeshwant Reddy Chillakuru, Shourya Munjal, Benjamin Laguna, Timothy L. Chen, Gunvant R. Chaudhari, Thienkhai Vu, Youngho Seo, Jared Narvid, Jae Ho Sohn

**Affiliations:** 1 Radiology & Biomedical Imaging, University of California San Francisco (UCSF), 505 Parnassus Ave, San Francisco, CA 94158 USA; 2 The George Washington University School of Medicine and Health Sciences, 2300 I St NW, Washington, DC 20052 USA; 3 Rice University, 6100 Main St, Houston, TX 77005 USA

**Keywords:** Natural language processing, Protocol, Automation, Neuroimaging

## Abstract

**Background:**

A systematic approach to MRI protocol assignment is essential for the efficient delivery of safe patient care. Advances in natural language processing (NLP) allow for the development of accurate automated protocol assignment. We aim to develop, evaluate, and deploy an NLP model that automates protocol assignment, given the clinician indication text.

**Methods:**

We collected 7139 spine MRI protocols (routine or contrast) and 990 head MRI protocols (routine brain, contrast brain, or other) from a single institution. Protocols were split into training (n = 4997 for spine MRI; n = 839 for head MRI), validation (n = 1071 for spine MRI, fivefold cross-validation used for head MRI), and test (n = 1071 for spine MRI; n = 151 for head MRI) sets. fastText and XGBoost were used to develop 2 NLP models to classify spine and head MRI protocols, respectively. A Flask-based web app was developed to be deployed via Heroku.

**Results:**

The spine MRI model had an accuracy of 83.38% and a receiver operator characteristic area under the curve (ROC-AUC) of 0.8873. The head MRI model had an accuracy of 85.43% with a routine brain protocol ROC-AUC of 0.9463 and contrast brain protocol ROC-AUC of 0.9284. Cancer, infectious, and inflammatory related keywords were associated with contrast administration. Structural anatomic abnormalities and stroke/altered mental status were indicative of routine spine and brain MRI, respectively. Error analysis revealed increasing the sample size may improve performance for head MRI protocols. A web version of the model is provided for demonstration and deployment.

**Conclusion:**

We developed and web-deployed two NLP models that accurately predict spine and head MRI protocol assignment, which could improve radiology workflow efficiency.

## Background

As the use of magnetic resonance imaging (MRI) has increased substantially in the past two decades, the variety and complexity in MRI protocols have grown rapidly [1, 2]. An accurate and systematic approach to protocoling can reduce errors as well as improve efficiency and patient safety [3]. However, manual protocoling can be a time-consuming task, accounting for 6.2% of a radiologist’s workday [4]. Moreover, neuroimaging protocols are especially complex, and less experienced radiology trainees and technicians are more likely to make protocol errors [5]. However, validated computerized decision support tools can improve practitioner performance and efficiency, while reducing workload burden [6, 7].

Natural language processing (NLP) is the application of machine learning models to classify and analyze free, or “natural”, text, such as clinical indications given in radiology protocols [8, 9]. NLP has been demonstrated to be effective at analyzing medical free text found in electronic medical records to improve radiology quality assurance, extract cancer characteristics, identify incidental findings, and improve protocol workflows [10–15]. However, the application of NLP in radiology is still relatively in its early stage, and NLP, as a field of study, consists of a wide-array of techniques and algorithms, each with its own strengths and weaknesses. Moreover, results are often specific to the data used in model building and evaluation. If the dataset contains simple cases, results will be artificially inflated, but if the dataset contains complex cases and heterogeneous protocoling standards (e.g. from different institutions), model performance may not reflect real-world performance.

Although some studies have shown promise in automating imaging protocols, NLP protocoling algorithms must be further tested because of both the rapid evolution of NLP techniques and the need for accurate protocol assignment in patient care [13, 15]. While prior studies of NLP in radiology have demonstrated promising results, in terms of accuracy, relatively little work has gone into understanding what factors influence a model’s classification success and, furthermore, what degree of harm may come from classification errors. Therefore, in this study, we develop and evaluate two algorithms to classify protocols for head and spine MRIs, respectively. In addition, we explore what words and phrases influence model decision making and perform a systematic error analysis of incorrectly classified protocols.

## Methods

### Data acquisition

**Fig. 1:**
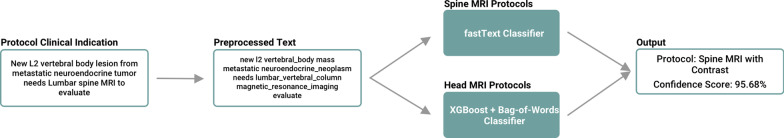
Overview of NLP pipeline. Overview of natural language processing (NLP) pipeline for automating spine and head MRI classifiers using an example protocol clinical indication. Clinical indication text is preprocessed to reduce non-essential information and group related terms together. Processed text is then transformed into a vector representation and passed through the appropriate model, which then outputs a protocol assignment and confidence score

This institutional review board approved, written informed consent-waived, and HIPAA compliant study collected de-identified protocol assignments and clinical indications from a single academic institution. Protocol assignments and clinical indications for MRIs of the spine and the head from January 2017 to January 2018 were obtained. These protocols were assigned as part of normal clinical workflow at our institution by trainees as well as attendings; this process includes the ability to prescribe or withhold contrast when the radiologist deems appropriate. MR-guided interventional radiology protocols and protocols without clinical history were excluded. Spine MRI protocols (n = 7139) were split into training (70%), validation (15%), and test (15%) sets. Because of relatively few samples, head MRI protocols (n = 990) were split into training (85%) and test (15%) sets, and fivefold cross validation was used in place of a validation set for hyperparameter tuning. Spine MRI data included protocols for cervical spine, thoracic spine, lumbar spine, and total spine each of which contained two protocol assignments: without contrast and with contrast. Head MRI data contained nine protocol assignments: routine MR brain without contrast (routine brain), routine MR brain with contrast (contrast brain), MR internal auditory canal (IAC), MR face with contrast, MR orbits with contrast, MR sella with contrast, magnetic resonance angiography (MRA), MR epilepsy, and temporomandibular joint (TMJ). Protocol clinical indications were preprocessed and passed to the appropriate algorithm (Fig. 1).

### Preprocessing

Clinical indications for each study are entered by referring clinicians in a free text field as to the indication for the ordered MRI. These clinical indications were automatically extracted from the electronic medical record (EMR) and de-identified. The algorithms did not use or have access to other elements or fields of the EMR. Clinical indications for each protocol were preprocessed with the following steps. Sentences were separated; dates and times were replaced with “DATE” and “TIME” respectively. Semantic-dictionary mapping was used to consolidate common terms [16]. For example, “no”, “rule out”, “r/o”, “absent” would all be mapped to “NEGEX” (i.e. negative expression). Next, radiology-specific terms were similarly mapped and grouped using RadLex [17]. For example, “magnetic resonance imaging” and “MRI” would both be mapped to “magnetic_resonance_imaging.” We then mapped the negative term to apply to all following words within the sentence. Therefore, “65 yo male. r/o hematoma and abscess” would be converted to “AGE male NEGEX_hematoma NEGEX_and NEGEX_abscess”. Because a bag-of-words-based model was used for head MRI clinical indications, this data had one additional preprocessing step: “term-frequency times inverse document-frequency” transformer was used to create a weighted vector representation of each word in a protocol [18].

### NLP models

FastText was used to classify spine MRI protocols, and XGBoost with bag-of-words was used for head MRI classification [19]. Both fastText and XGBoost bag-of-words were considered for both spine and head MRI protocol classifications. The current system was chosen as fastText had higher performance on spine MRI validation data and XGBoost higher on head MRI validation data. Because of the limited, unique corpus and mapping with RadLex., we believed that classical and simpler language models would achieve similar performance compared to more advanced methods, such as transformers. FastText is a lightweight machine learning software, from which we utilize a classification algorithm that represents words as character components. XGBoost is a gradient-boosting machine learning software, from which we utilize a tree-based boosting algorithm. Both fastText and XGBoost techniques have achieved high performance in prior studies [19, 20]. Grid search was used to fine-tune hyperparameters on validation data. FastText was trained using softmax loss over 6 epochs with a word vector size of 100, learning rate of 0.1, and bigrams (wordNgram = 2). XGBoost was trained with negative log loss, 100 tree estimators, a learning rate of 0.1, 80% subsampling per tree, and a max tree depth of 8 and required a 1 point loss reduction to further partition nodes (gamma = 1). Both XGBoost and fastText would output a confidence score between 0 and 1 for each protocol, representing the likelihood that a given clinical indication should be assigned to that protocol.

### Model evaluation & error analysis

Model performance was assessed by calculating the overall accuracy and receiver operating characteristic area under the curve (ROC-AUC) for brain and spine MRIs respectively. In order to calculate the word importance score in model decision making, each unique preprocessed word in our dataset was entered into the model as a separate input to calculate a word importance score. This importance score was used to identify which individual words had the largest influence on model classification of a protocol as routine, contrast, or other. The “other” category applies only to the head MRIs and includes IAC, sella, epilepsy, face, MRA, orbits, and TMJ protocols. Additionally, we plotted the distribution of correct and incorrect predictions to understand whether low confidence scores truly reflect uncertainty in the model prediction. We also manually reviewed a select number of incorrectly classified protocols to identify any systematic errors. Errors were classified as true errors, ambiguous cases, or incorrect ground truths by a board-certified radiologist and a neuroradiology fellow. Preprocessing, model development, and evaluation was done in Python 3.6 (Python Software Foundation, Delaware, United States). Data visualization and descriptive statistics, specifically Chi-square test and Kolmogorov–Smirnov test, were done in R (R Foundation for Statistical Computing, Vienna, Austria). The training and test set evaluation code is available at https://bit.ly/2ytg4FL.

### Web deployment

We created a live demonstration of the spine and head MRI protocol models as a python web application, using the Flask web framework and deployed on the cloud application hosting service, Heroku.

## Results

### Spine MRI

**Fig. 2:**
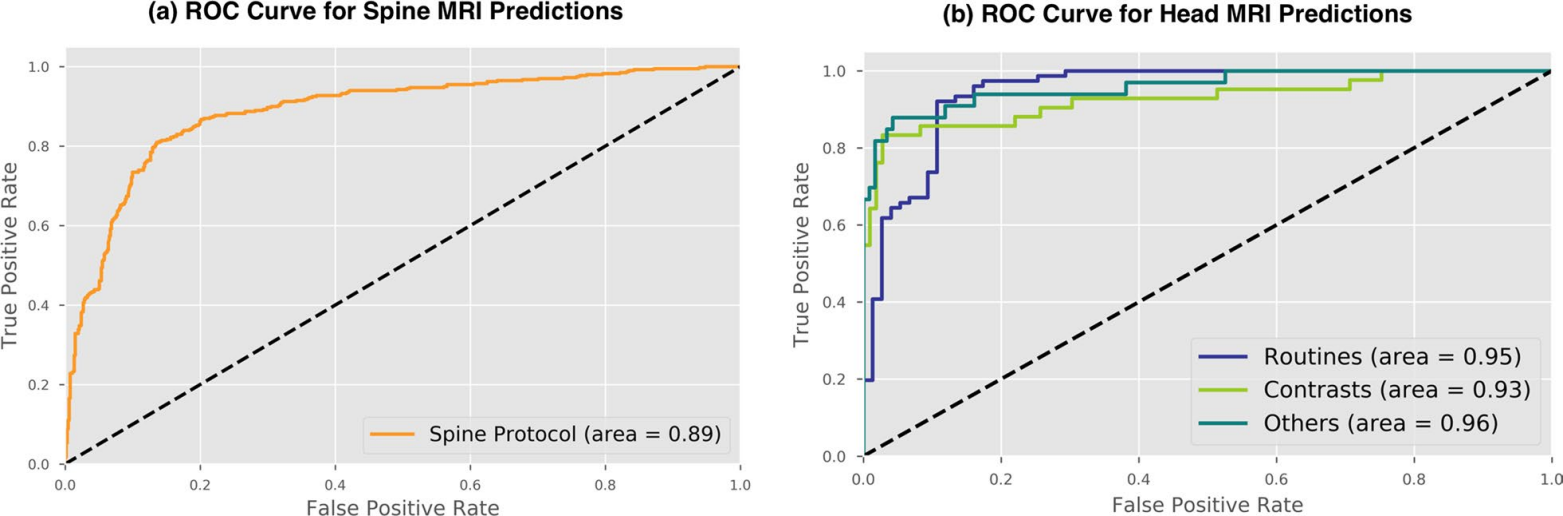
Receiver operating characteristic curves. Receiver Operating Characteristic (ROC) curves for spine and head MRI predictions. **a** Spine protocol achieved a ROC area under the curve (ROC-AUC) of 0.8873 for differentiating between contrast and non-contrast spine MRI. **b** Head MRI ROC-AUC was calculated as one vs. other categories for brain MRI routine without contrast (Routines), brain MRI with contrast (Contrasts), and Others (epilepsy, IAC, face, MRA, orbits, sella, and TMJ) and achieved a performance of 0.9463, 0.9284, and 0.9569, respectively

**Fig. 3:**
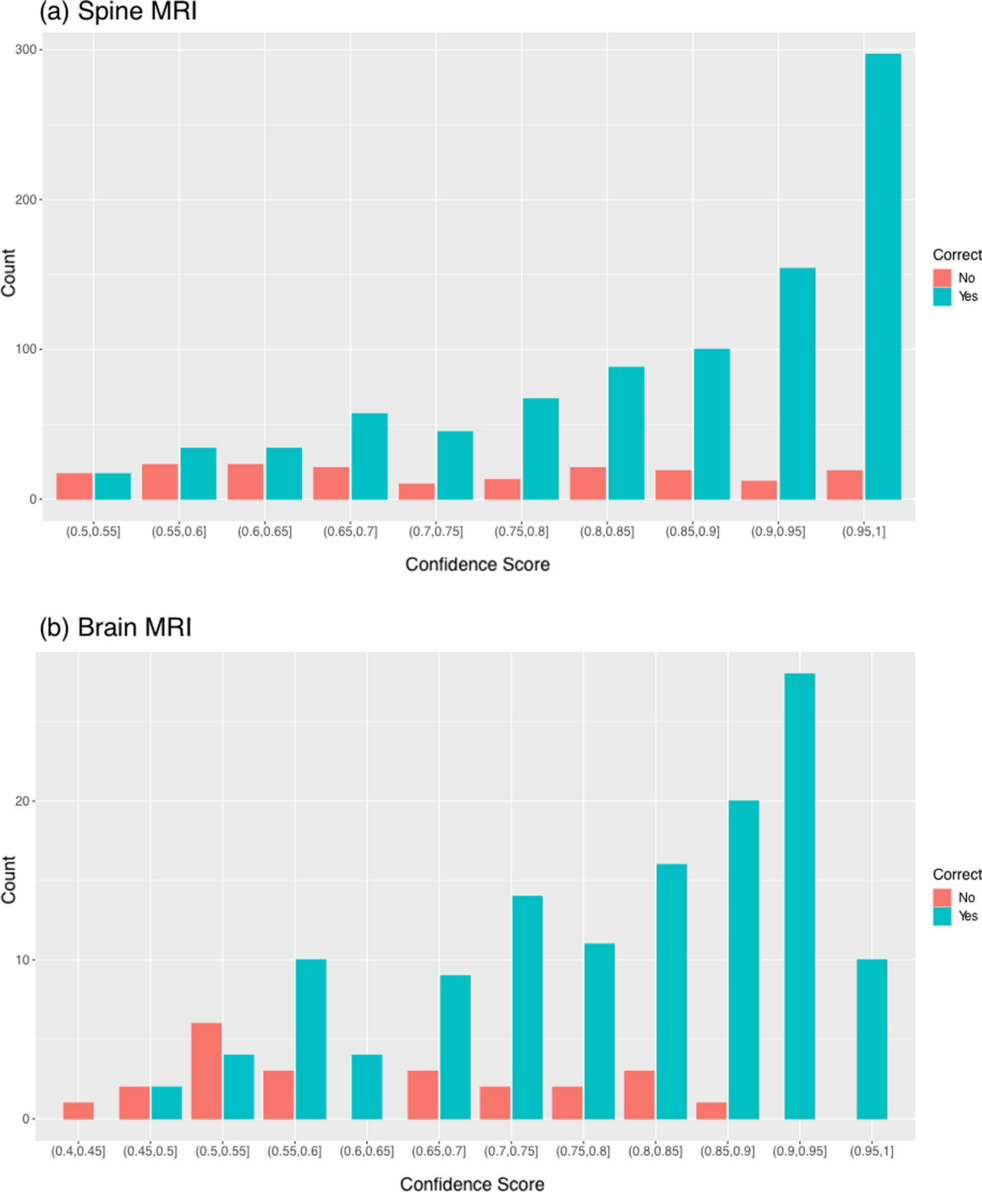
Confidence score of correct and incorrect predictions. A histogram of confidence scores for correct and incorrect protocol predictions. Confidence score obtained from model output on scale of 0–1. **a** For spine MRI prediction, confidence score distribution of incorrect predictions differed significantly from correct predictions (Kolmogorov–Smirnov test, *p* < 0.001). Predictions were skewed towards high confidence, while incorrect predictions were more uniformly distributed. **b** For brain MRI predictions, confidence score distribution of incorrect predictions differed significantly from correct predictions (Kolmogorov–Smirnov test, *p* < 0.001). Correct predictions were skewed towards high confidence

Spine MRI protocols were classified as either contrast or routine. Training (n = 4997), validation (n = 1071), and test (n = 1071) sets were each composed of similar portions of contrast protocols (38.54%, 36.32%, 37.25%, respectively, Chi-square *p* = 0.3399). On the test set, ROC -AUC was 0.8873 (Fig. 2a), and overall classification accuracy 83.38% at a confidence score threshold of 0.5. Precision for contrast administration on the test set was 80.27%, and recall was 73.43%. Protocols predicted as routine made up a majority of incorrect predictions (59.55%). Figure 3a shows that correct spine MRI protocol predictions are skewed to high confidence predictions, while incorrect predictions are more evenly distributed across confidence groups.

Words most associated with contrast and routine protocol assignment are detailed in Table 1. Words associated with cancer and inflammatory conditions had the strongest association for spine MRI with contrast administration. With the exception of “angiosarcoma” and “female,” degenerative conditions and structural problems were most associated with routine spine MRI.

**Table 1:** Words important for algorithmic protocol assignment

Position	Spine MRI Routine (importance score)	Spine MRI Contrast (importance score)	Head MRI Routine (importance score)	Head MRI Contrast (importance score)	Head MRI Other (importance score)
1	Stenosis (1.0000)	Mass (1.0000)	Stroke (0.9183)	Brain (0.9308)	Aneurysm (MRA: 0.9360)
2	Scoliosis (1.0000)	Resection (1.0000)	Non (0.9166)	Meningioma (0.8875)	Seizure (Epilepsy: 0.8911)
3	Disc (1.0000)	Infection (0.9992)	Severe (0.9050)	magnetic_resonance_angiography (0.5394)	negex_pituitary_gland (Sella: 0.7545)
4	Fall (1.0000)	Metastases (0.9989)	Memory (0.9005)	negex_brain (0.3836)	Hearing (IAC: 0.6439)
5	Herniation (1.0000)	Abscess (0.9983)	magenetic_resonance_imaging (0.8837)	Mass (0.3678)	negex_aneurysm (MRA: 0.4070)
6	Female (0.9999)	ependeymal_tumor (0.9976)	Cognitive (0.8821)	Evaluate (0.3122)	internal_auditory_canal (IAC: 0.3482)
7	myelomalacia (0.9999)	breast_cancer (0.9958)	Stability (0.8809)	pituitary_gland (0.3064)	Seizures (Epilepsy: 0.2725)
8	intervetebral_disc_degeneration (0.9998)	prostate_cancer (0.9948)	hx (0.8774)	Fluorine (0.3054)	galactorrhea (Sella: 0.2409)
9	Cervicalgia (0.9998)	Radiation (0.9943)	Weakness (0.8764)	Mets (0.2862)	pituitary_gland (Sella: 0.2367)
10	angiosarcoma (0.9997)	Myelitis (0.9934)	Months (0.8739)	Metastatic (0.2741)	Neuralgia (Face: 0.1571)

All incorrectly predicted protocols (n = 179) in the spine MRI test set were manually analyzed and 10 representative examples were included Table 2. Errors occurred at a variety of confidence score levels. High confidence errors (confidence score ≥ 0.90) appeared to occur when the algorithm prediction was a more appropriate protocol than the ground truth label, given the clinical indication text (e.g. Position 2 and 3; Table 2). Among the 31 high confidence errors in the entire test set, only 2 were truly incorrect (i.e. model error), and an additional 3 were ambiguous, requiring further information for a radiologist to make the appropriate protocol assignment. These 5 cases were classified as routine when the ground truth was contrast. Medium confidence errors (confidence score: 0.60–0.80) appear to be true model errors in prediction. They appeared to often occur in contrast protocols when the patient has a primary non-contrast issue, such as degenerative or structural disease, but had a secondary contrast issue, such as history of cancer (Positions 46, 47, 81, and 128; Table 2). Low confidence errors (confidence score: < 0.60) were more ambiguous cases upon radiologist review and could be classified as either contrast or non-contrast (Positions 167 and 168; Table 2).

**Table 2:** Spine MRI model error analysis

Position^a^	Correct protocol	Predicted protocol (confidence score)	Raw clinical history	Radiologist assessment
2	Contrast	Routine (0.9989)	AGE female with right lumbar radiculopathy	Not an error
46	Contrast	Routine (0.8591)	AGE female with lower back pain and right hip and leg radiculopathy. High risk breast cancer patient	True error
81	Contrast	Routine (0.7603)	Left sided sciatica. History of prostate cancer	True error
128	Contrast	Routine (0.6262)	AGE female patient with personal history of breast cancer with foot tingling	True error
168	Contrast	Routine (0.5356)	Status post abdominal wall explantation with history of multiple epidural anesthesia attempts. Now with headache and back pain	Can be either routine or contrast
3	Routine	Contrast (0.9961)	Status post resection of cerebellopontine angle mass	Not an error
47	Routine	Contrast (0.8569)	Coincident traumatic brain and spinal cord injury in DATE	True error
82	Routine	Contrast (0.7610)	Evaluation for recurrent herniation at L4-L5 history of L4-L5 discectomy on DATE	Can be either routine or contrast
129	Routine	Contrast (0.6246)	Status post L2-S1 posterior spinal fusion completed by hardware infection admitted for surgery	Not an error
167	Routine	Contrast (0.5364)	Status post thoracic fusion and removal of hardware. Patient complains of severe mid thoracic pain and radiation to the front. Please evaluate for thoracic cord impingement or nerve root impingement	Can be either routine or contrast

^a^The position refers to the ordered rank when all 178 incorrectly predicted spine MRI protocols are ordered from most incorrect (high confidence score) to least incorrect (low confidence score)

### Head MRI

Head MRI protocols were classified into 9 different protocol assignments. In the training data (n = 839), the most common protocols were routine brain without contrast (n = 410) and routine brain with contrast (n = 202). The other 7 protocols, making up a minority of the training data, were epilepsy (n = 63), sella (n = 46), IAC (n = 45), MRA (n = 38), face (n = 25), orbits (n = 8), TMJ (n = 2). The head MRI test set (n = 151) contained a similar distribution of protocol categories compared to the training set (Fisher Exact *p* = 0.2855).

Overall accuracy of the head MRI protocol on the test set was 85.43%. ROC-AUC, calculated as one category versus the rest, for routine protocols was 0.9463; contrast was 0.9284; and other specialized protocols combined (epilepsy, IAC, face, MRA, orbits, sella, and TMJ) was 0.9569 (Fig. 2b). Precision for contrast brain protocol was 96.77%, and recall was 72.43% at a confidence score threshold of 0.5.

Words most associated with routine protocol assignment were generally related to strokes, chronic conditions (e.g. a history including words related to chronicity, such as “months” and “hx”), and cognition (Table 1). Words most associated with contrast brain MRI protocols were generally suggestive of cancer (Table 1). Words associated with other specialized protocols were often specific to that protocol, such as “aneurysm”, “internal_auditory_canal”, “seizure”, and “galactorrhea” for MRA, IAC, epilepsy, and sella protocols, respectively (Table 1).

Twenty four incorrectly predicted protocols in the head MRI test set underwent manual error analysis in order to understand potential causes of misclassification and 10 representative cases are shown in Table 3. A majority of examples were true errors, and 82.61% of errors occurred due to overprediction of routine brain MRI protocol. There were no high confidence errors (confidence score ≥ 0.90) in the head MRI test set.

**Table 3:** Head MRI model error analysis

Position^a^	Correct protocol	Predicted protocol (confidence score)	Raw clinical history	Radiologist assessment
1	IAC	Routine Brain (0.8837)	MRI to rule out any CPA/retrocochlear masses that might be causing her vestibular symptoms and hearing loss. per EN: MRI to rule out any CPA/retrocochlear masses that might be causing her vestibular symptoms and hearing loss	True error
3	TMJ	Routine Brain (0.8313)	Does this patient have TMJ arthritis and/oe anterior disc displacement w/o reduction. Consistent TMJ pain not resolved by massage, NSAIDS or muscle relaxants. Feels like something is blocking her mouth when she opens. Pain is extreme	True error
5	ContrastBrain	Routine Brain (0.7986)	interval assessment for Multiple sclerosis. follow up MRI in next 1–2 months	Contrast preferred, Routine may be ok
7	Orbits	Routine Brain (0.7464)	Optic nerve pallor. Likely longstanding optic nerve pallor with visual field defect. R/o tumor or compression	True error
9	ContrastBrain	Routine Brain (0.6947)	f/u glioblastoma multiforme. GBM, s/p surgery, RT, on chemo; ?progression on last MRI—reviewed at FACILITY Neuro Onc—rec 2 month f/u as pt clinically stable	True error
11	Contrast Brain	Routine Brain (0.6785)	CNS lymphoma. AGE M with HIV and relapsed primary CNS lymphoma receiving WBRT will complete on DATE. Needs post-radiation scan	True error
13	Face	Routine Brain (0.5824)	r/o facial nerve abnl. this AGE woman has Turner's syndrome and has now had N recurrent episodes of "Bell's palsy" sequentially involving both sides of her face. query compression in facial canals vs other base of skull abnl involving her facial nerves bilaterally patient also experienced tongue and perioral numbness (not clearly just loss of taste) so evaluation of course of bilat V3 also appreciated	True Errorl
15	IAC	Routine Brain (0.5485)	Request MRI IAC protocol to eval new unilateral tinnitus; patient has symmetric SNHL, though subjectively worse on left. New unilateral (left) tinnitus over last six months; also with symmetric SNHL on audio (including symmetric word recognition scores), however hearing is subjectively worse on left	True error
17	Routine Brain	Contrast Brain (0.5247)	thinking changes and new headache	True error
19	f	Contrast Brain (0.5131)	Evaluate for structural etiology of HA. AGE woman with severe migraines precipitated by aura of left arm numbness, then with pounding right-sided HA and nausea. Previously evaluated by Neuro, had normal brain CT and recommended advancing imaging if severe HA returns. Symptoms returned periodically since once month ago, requesting MRI for further evaluation of structural cause	True error

^a^The position refers to the ordered rank when all 23 incorrectly predicted head MRI protocols are ordered from most incorrect (high confidence) to least incorrect (low confidence)

### Live demo

We provide a simple demonstration of our automated brain and spine MRI protocoling models on https://bit.ly/3d7Lnow.

## Discussion

### Overview

We developed two NLP models to automatically determine spine and head MRI protocol assignments in order to potentially improve clinical workflow efficiencies. While accurate protocoling is vital for patient care and for delivery of cost-efficient healthcare, protocoling can be time consuming and account for up to 6.2% of a radiologist’s work hours [3, 4]. Our models achieved strong performances of 83.38% and 85.43% accuracy on spine and head MRIs, respectively. Moreover, an analysis of word importance for each prediction showed that the models were identifying clinically relevant components from the clinical indication text. For example, words associated with cancer were ranked high on word importance for contrast protocols.

### Analysis of protocol automation models

Brain and spine MRI guidelines recommend contrast administration generally for infectious, neoplastic, or inflammatory processes [21]. Manual assessment of the MRI classifiers found that words associated with these conditions had high importance scores for administration of contrast. Similarly, words associated with structural issues for spine MRI and cognitive, stroke, and mental status issues for head MRI were more strongly associated with routine brain protocol without contrast (Table 1). This demonstrates that NLP models can adapt well to domain specific problems in medicine, even with the relatively small corpus and data size used in this study. For comparison, NLP algorithms used in general consumer products are often trained on massive texts, such as the entirety of Wikipedia. However, while this model is learning the underlying relationship between clinical indication and protocol in many cases, it is still relying on statistical associations, as is evident by the word “female” having a high importance score for routine spine MRI protocols. These statistical associations are a critical pitfall in the application of algorithms to medical decisions as they may reinforce biases present in the training dataset. Biases in training data could lead to further marginalization of minority groups, exacerbation of healthcare disparities, and errors in classification [22, 23]. NLP models are known to reflect socioeconomic biases inherent in human free-text [24]. The association made by the spine MRI model with “female” raises the possibility that clinical protocoling is not immune from these biases. Future models could incorporate more advanced techniques, such as variational autoencoders, to reduce bias in machine learning algorithms [25].

The quality of input data is vital to developing an accurate model. During manual error analysis of spine MRI models, several protocols had seemingly incorrect ground-truth labels. While this may be a mistake on the radiologist who was protocoling in real-time, it is more likely that the radiologist had access to other information (the complete EMR) not directly stated in the clinical history within our de-identified dataset. An additional issue common to training sets in general is unbalanced data. The head MRI model demonstrated how unbalanced data can lead to systematic errors, as the head MRI data contained only a few training examples of the specialized protocols (IAC, MRA, epilepsy, sella, face, orbits, and TMJ). Word importance analysis demonstrated that, despite having a small number of samples, the model was recognizing some keywords, such as internal_auditory_canal for IAC. However, these associations were not strong enough, and the algorithm failed to recognize some obvious specialized protocols. Additionally, a majority of the brain training data was routine protocols. These two factors—few examples of specialized protocols and unbalanced training data—led to systemically incorrectly predicting many protocols as routine. Nearly all head MRI errors (82.61%) were made by incorrectly predicting protocols as routine brain. However, this issue can be rectified in the future by training with data that has an artificially inflated number of specialized protocols.

### Error analysis

Our analysis confirms that protocols with high confidence score (≥ 0.90) predictions are much more likely to be correct than low confidence scores (Fig. 3). Furthermore, no high confidence errors existed in the head MRI test set. Of the 31 high confidence errors in the spine MRI test set, only 2 were truly incorrectly classified and 3 more lacked sufficient information in the clinical indication text. All 5 cases were incorrectly labeled routine instead of contrast, which is a more favorable error than inappropriate contrast administration. Thus, an important feature of our models is that in high confidence errors the models avoided suggesting unnecessary administration of contrast. Based on predictions on the test sets, 10% of spine MRI cases and 13% of head MRI cases would have to return for contrast administration or a specialized protocol. However, if only high confidence predictions (≥ 0.90), which account for 40% of test set predictions, were automated, then only 5 out of 482 high confidence spine MRI cases (1%) and no brain MRI cases would have to return to for contrast administration or a specialized protocol.

### Clinical integration workflow

**Fig. 4:**
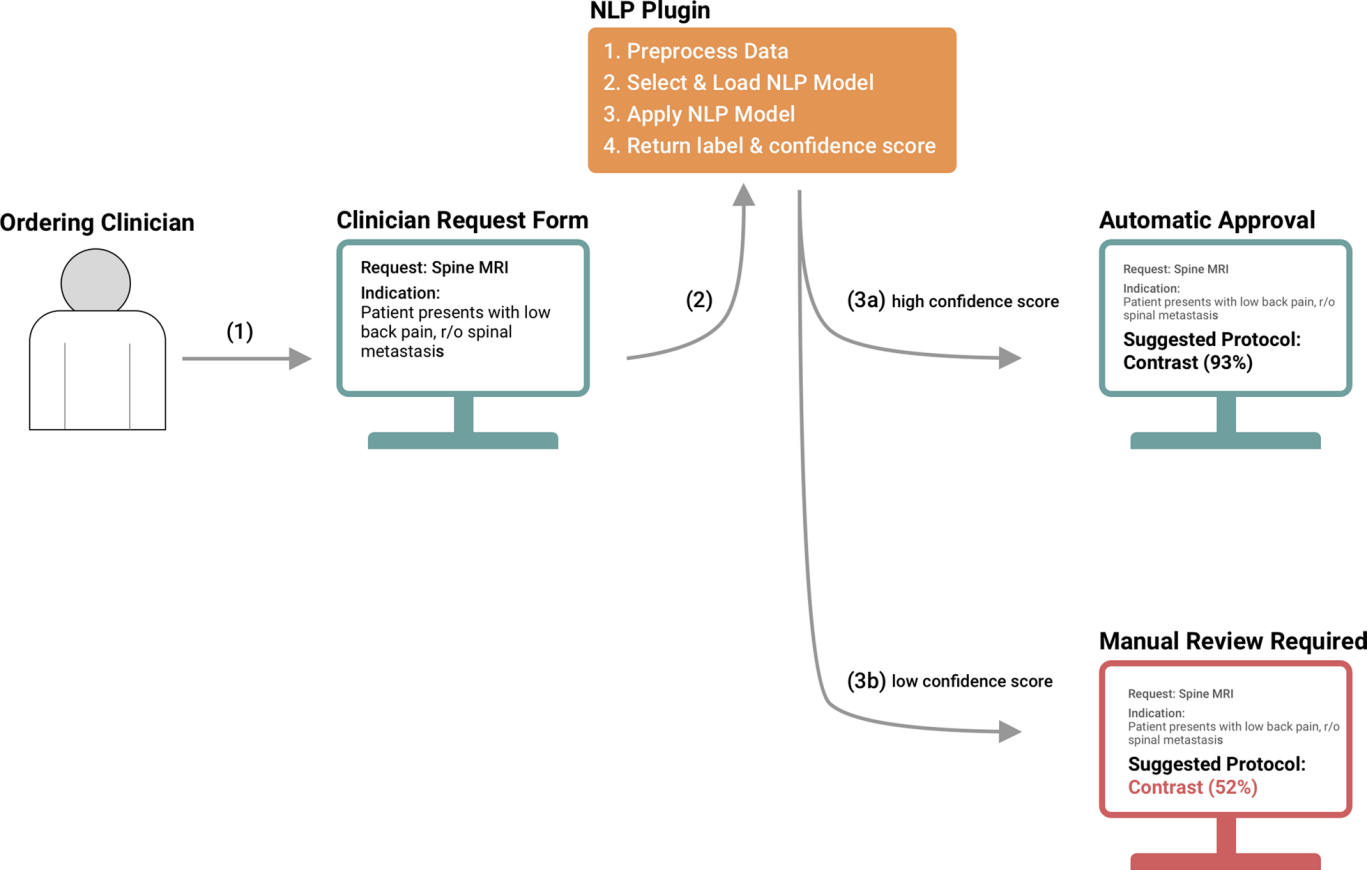
Integration of automated protocoling. Suggested integration of automated protocoling. (1) The ordering clinicians submits an MRI request form. (2) Request form is queried by NLP plugin and analyzed. (3a) High confidence scores above 0.90, and patients without contraindications are classified for automatic approval. (3b) Otherwise, patients with complex contraindications or lower confidence scores are sent for manual review with the algorithmic suggestion

While we have demonstrated the potential to use NLP for MRI protocoling, true clinical integration must rely on the convergence of several important factors. A multi-institution training data set must be created to develop a generalizable algorithm. Ideally, this dataset would conform to a single set of protocoling guidelines and will be updated, along with the machine learning model, as guidelines change. While clinical decision support tools have been shown to improve healthcare process measures [6], automated protocoling must be implemented in a way to overcome default bias (accepting the algorithm recommendation when it is incorrect) and overconfidence bias (ignoring algorithmic recommendation when it is correct) [22]. The most likely clinical integration scenario would be to only permit automated acceptance of high confidence scores (Fig. 4). Because 40% of our test set predictions had a confidence score above 0.90 (Fig. 3), we can expect automatic approval of only high confidence predictions to reduce spine and brian MRI protocol workload by approximately 40%. Future implementation studies will be needed to test this hypothesis. Moreover, integration of new technology into a workflow must include input from and testing with end users. As exemplified with the electronic medical record, technology aimed at “efficiency” can lead to increased time spent documenting if not carefully integrated [23]. Implementations should be flexible enough for inter-departmental workflow variations, but consistent enough to not overburden hospital information technology teams with maintenance. We suggest developing a simple plugin to integrate NLP protocol models with the existing workflow (Fig. 4). Additionally, clinical data can be pulled from the electronic medical record to flag cases with contraindications to contrast for manual review. Most importantly, a systematic quality assurance review should be applied to categorize errors as ordering clinician error, trainee error, inherent protocol error, or predictive NLP model error and to investigate their cause [5].

### Limitations

This study faces several limitations. First, NLP models determine protocol assignment by word and word-context relationships. However, these can lead to unintended use of non-medically relevant, human biases hidden in the data. Second, our head MRI protocol data lacked sufficient sample size on more specialized protocols. Third, since this data comes from a single academic institution, its generalizability to other institutions should be tested before implementation. Training with multi-institutional data may be required to overcome generalizability issues. Despite these limitations, our model still achieved strong performance, and future NLP projects can work to address these short-comings using additional preprocessing steps, collecting data from multiple institutions, and ensuring specialized protocols are adequately represented in the data.

## Conclusion

NLP can be used to effectively automate protocoling of spine and head MRIs. Our analysis revealed that the NLP models could learn relevant associations between disease states and protocol assignments. Future research includes assessment of gains in workflow efficiency from automated protocoling as a clinical decision support tool.

## Data Availability

The datasets generated and/or analysed during the current study are not publicly available due concerns for confidentiality as some clinical indication texts contain patient information. However, the model training and test code is available at https://bit.ly/2ytg4FL, and a live demo is available at https://bit.ly/3d7Lnow in an effort to promote transparency.

## Abbreviations

AUC: Area under the curve
EMR: Electronic medical record
IAC: Internal auditory canal
MRA: Magnetic resonance angiography
MRI: Magnetic resonance imaging
NLP: Natural language processing
ROC: Receiver operating characteristic
TMJ: Temporomandibular joint
